# Bicuspid aortic valve stenosis is characterized by increased angiogenesis, inflammation, and a higher valvular-to-systemic calcification ratio than tricuspid aortic valve stenosis

**DOI:** 10.1007/s00395-026-01191-8

**Published:** 2026-06-27

**Authors:** Alexander Brückner, Adrian Brandtner, Sarah Rieck, Hannah Billig, Werner Masson, Anna Weber, Farhad Bakhtiary, Wilhelm Röll, Christoph Bourauel, Frank A. Schildberg, Baravan Al-Kassou, Sebastian Zimmer, Daniela Wenzel, Bernd K. Fleischmann

**Affiliations:** 1 Institute of Physiology I, Medical Faculty, University of Bonn, Bonn, Germany; 2 Department of Internal Medicine 2, University Hospital Bonn, Bonn, Germany; 3 Department of Orthopedics and Trauma Surgery, University Hospital Bonn, Bonn, Germany; 4 Department of Oral Technology, University Hospital Bonn, Bonn, Germany; 5 Department of Cardiac Surgery, University Hospital Bonn, Bonn, Germany; 6 Institute of Physiology, Department of Systems Physiology, Medical Faculty, Ruhr University of Bochum, Bochum, Germany

**Keywords:** Aortic valve stenosis, Gene expression, Calcification

## Abstract

Calcific aortic valve stenosis is the most common valvular heart disease requiring treatment. Although both tricuspid (AVS) and bicuspid (bAVS) aortic valve stenoses become calcified and functionally impaired in advanced stages, the pathophysiology of these conditions remains unclear. We investigated this using a multitechnology approach on explanted AVS, bAVS, and aortic regurgitation (AR) control valves. Because of technical limitations in processing heavily calcified aortic valve tissue, we established Kawamoto’s film method for human aortic valve tissue, enabling the production of well-preserved cryosections and high-quality immunostainings. Both bulk RNA-seq analysis and immunostainings revealed that angiogenesis, inflammation, and calcification are key features distinguishing bAVS from AVS. In fact, we found that angiogenic genes and CD31^+^ cells, as well as inflammatory genes and CD45^+^ cells, are significantly elevated in bAVS. The most striking difference between bAVS and AVS was the prominent expression of specific genes involved in tissue calcification, such as matrix metallopeptidase 12 (*MMP12*), dentin matrix acidic phosphoprotein 1 (*DMP1*), and proteoglycan 4 (*PRG4*), along with approximately 1.7-fold increased calcification as shown by micro-CT and von Kossa staining analysis in bAVS. These findings were corroborated in a retrospective analysis of 1108 AVS and bAVS patients who underwent transcatheter aortic valve implantation (TAVI). The bAVS patients exhibited significantly stronger aortic valve calcifications (1.6-fold) but a significantly lower vascular calcification burden. These data further suggest that AVS and bAVS are distinct disease entities, with bAVS exhibiting increased local inflammation, angiogenesis, and calcification, findings that may guide future therapeutic strategies.

**Supplementary Information:**

The online version contains supplementary material available at 10.1007/s00395-026-01191-8.

## Introduction

The aortic valve is composed of three distinct layers: the fibrosa, spongiosa, and ventricularis, which contain valve interstitial cells (VICs) and an extracellular matrix. The leaflets are covered on both the aortic and ventricular sides by endothelial cells (VECs) of the valve, which regulate hemostasis, barrier function, and immune responses [19]. Aortic valve stenosis is a common and severe condition in the elderly. It often remains silent for extended periods but typically requires invasive treatment when symptoms arise [44], ideally before significant impairment of coronary perfusion, LV hypertrophy, and ensuing heart failure develop [18]. Tricuspid aortic valve stenosis (AVS) is the most prevalent form, characterized by diffuse calcification of all three leaflets and typically affecting individuals in their 70s and 80s. In contrast, individuals with a bicuspid aortic valve, the most common congenital heart defect, have only two cusps of the aortic valve and are at risk for the early development of bicuspid aortic valve stenosis (bAVS) between 40 and 60 years of age [5].

The pathogenesis of AVS is thought to involve initiation and propagation stages [29, 43]. The former is characterized by endothelial cell dysfunction resulting from mechanical and shear stress, followed by lipid deposition and immune cell infiltration [39]. In addition, adverse stimuli, such as oxidative stress, dyslipidemia, and inflammation, are thought to trigger the transdifferentiation of VECs into VICs via endothelial-to-mesenchymal transition (EndoMT) [8, 26, 30], contributing to disease progression. During the propagation phase, VICs transdifferentiate into myofibroblastic and osteoblastic phenotypes, driven by factors and cytokines secreted by immune cells [37]. For instance, myofibroblastic differentiation increases expression of alpha-smooth muscle actin (α-SMA), vimentin, and smooth muscle myosin. Calcifications in aortic valves involve proteins such as osteopontin, a secreted phosphoprotein expressed by osteoblasts that plays a key role in biomineralization and bone remodeling. Elevated plasma osteopontin levels are associated with AVS, suggesting a functional role in modulating the disease [38, 40, 54]. Thus, although detailed mechanisms remain unclear, the pathophysiology of AVS appears to be associated with age-related degenerative changes and systemic risk factors typical of atherosclerosis. In contrast, bAVS is most likely caused by genetic predisposition and driven by abnormal hemodynamic forces resulting from congenital valve fusion, which accelerate valve degeneration and calcification [4]. Interestingly, calcification patterns are suggested to differ between AVS and bAVS [15], reflecting variations in valve morphology, biomechanics, and underlying pathophysiology. In an earlier study, we distinguished between the two disease entities by showing side-specific differences in VECs in AVS but not in bAVS [6]. Additionally, bAVS is often associated with aortopathy, characterized by dilation of the ascending aorta, and may coexist with connective tissue disorders or genetic predispositions that influence calcification [48].

Although advances in heart surgery and transcatheter aortic valve implantation (TAVI) have greatly improved treatment options for AVS and bAVS, causal therapy remains elusive [35]. The apparent differences in phenotypic presentation and clinical parameters between AVS and bAVS may suggest that they are distinct disease entities, underscoring the need for further investigation into their underlying molecular mechanisms. However, technical shortcomings have significantly impacted the histomorphological processing of calcified aortic valves, as the routinely used decalcification step disrupts tissue architecture and integrity [11, 17, 36, 39]. Therefore, we adapted Kawamoto’s film method for processing and sectioning human calcified aortic valves [24, 25] and combined immunostaining with in-depth bulk RNA-seq analysis and a retrospective analysis of a large cohort of TAVI-treated AVS and bAVS patients. Our data showed clear differences between AVS and bAVS, with bAVS exhibiting enhanced inflammation, angiogenesis, and pronounced localized calcification, in contrast to the more systemic vascular calcification observed in AVS. These findings are consistent with clinical data supporting distinct disease mechanisms and underscore the need for tailored therapeutic approaches.

## Methods

### Harvesting of the valves

Aortic valves were collected directly in the operating room after explantation from patients undergoing surgical aortic valve replacement at the University Hospital Bonn, Germany. The valves were stored in sterile 0.9% (w/v) sodium chloride solution (Fresenius Kabi, Bad Homburg, Germany) and processed on ice.

Based on the clinical data, the calcified valves were classified as bAVS and AVS stenoses, respectively. As non-calcified controls, valves from patients suffering from aortic regurgitation (AR) were used. In total, 75 valves (26 AVS, 32 bAVS, and 17 AR) were collected and used for bulk RNA-seq or immunofluorescence staining. Patient specifics from this surgical aortic valve replacement cohort (SAVR cohort) are summarized in Supplemental Table 1.

### Tissue fixation

Valves were fixed in 4% (w/v) paraformaldehyde solution (Sigma-Aldrich, Taufkirchen, Germany) for 24 h and washed three times for 5 min in PBS (Gibco, ThermoFisher Scientific, Darmstadt, Germany). Afterward, valves were embedded in SCEM (Section-lab Co. Ltd., Yokohama, Japan).

### Cryosectioning

Non-calcified and calcified valves were sectioned using a conventional cryosectioning method without cryofilm and with/without prior decalcification. Decalcification of calcified valves was performed in 10% EDTA solution for 72 h at 4 °C, followed by incubation in 20% sucrose for 12 h at 4 °C with washing steps with PBS in between. The temperature of the cryostat and sample holder was adjusted to − 22 °C and − 20 °C, respectively. Sections were cut with a feather microtome blade R35 (stainless steel; pfm medical GmbH, Cologne, Germany) with 10 and 15 µm thickness for immunofluorescence and histological stainings, respectively. Cryosections were transferred from the anti-roll plate to HistoBond® microscope slides (Marienfeld, Lauda-Königshoven, Germany) for further staining and analysis.

### Cryosectioning with adapted Kawamoto’s film method

A tungsten carbide blade (SL-T30UF; Section-lab Co. Ltd., Yokohama, Japan) was used to cryosection the calcified valves. The temperature of the cryostat and the sample holder was adjusted to − 30 °C and − 35 °C, respectively. Sections were cut at 10 µm and 15 µm thickness for immunofluorescence and histological stainings, respectively. The cryofilm [Type 3C(16UF), Section-lab Co. Ltd., Yokohama, Japan] was mounted to the trimmed sample block with the fitting tool according to the instructions of the manufacturer [24]. The specimen was slowly cut at a constant speed.

### Immunofluorescence staining

Immunofluorescence staining was performed at room temperature. Cells were permeabilized in PBS using 0.2% (v/v) Triton-X100 (Sigma-Aldrich, Taufkirchen, Germany). After washing and incubation with 5% (v/v) donkey serum (Jackson ImmunoResearch, Ely, UK) in PBS for 30 min, cells were stained with primary antibodies (anti-CD31 (1:400), kindly provided by Prof. Dr. Newman, University of Wisconsin; anti-CD31 (1:100), BioGenex #MU241-UC; anti-α-SMA (1:800), Sigma-Aldrich #A5228; anti-Osteopontin (1:100), abcam #ab69498; anti-Vimentin (1:200), Merck #AB5733; anti-CD45 (1:200), Santa Cruz Biotechnology #sc-1187; anti-Albumin (1:100), and Invitrogen #MA5-29,022) in 5% (v/v) donkey serum in PBS for 3 h. Secondary antibodies (Cy3- and Cy5-conjugated anti-rabbit, Cy3- and Cy5-conjugated anti-mouse IgG1, Cy3- and Cy5-conjugated anti-mouse IgG2a, Cy3- and Cy5-conjugated anti-chicken; all from Jackson ImmunoResearch, Ely, UK and diluted 1:400) were applied in Hoechst 33,342 (1 µg/ml; Sigma-Aldrich, Taufkirchen, Germany) for 1 h. Between and after staining with primary or secondary antibodies, the cryosections were washed 3 × in PBS. Aqua Polymount embedding medium (Polyscience, Warrington, USA) was used to mount cryosections between a HistoBond® microscope slide (Marienfeld, Lauda-Königshoven, Germany) and a cover glass to enable oil immersion microscopy. The highest image quality was achieved by combining an oil immersion objective with ApoTome-based image acquisition (Supplemental Fig. 2A,B).

### Quantification of vessels within aortic valves

CD31 immunofluorescence stainings were used to quantify vessels within aortic valves. Vessel-like structures were counted when at least two CD31^+^ cells and a lumen were identified. The number of vessels was normalized to the soft-tissue area of the valve without calcification. Area measurement was performed using ImageJ.

### Movat’s pentachrome staining

Cryosections were washed once in ddH_2_O before staining for 10 min in 1% (w/v) alcian blue (Carl Roth, Karlsruhe, Germany) solution containing 1% (v/v) glacial acetic acid (Carl Roth, Karlsruhe, Germany). After washing in H_2_O, sections were incubated for 1 h in alkaline ethyl alcohol (10% (v/v) ammonium hydroxide (Sigma-Aldrich, Taufkirchen, Germany) in ethanol (96%) (AppliChem GmbH, Darmstadt, Germany)). Afterward, sections were hydrated in H_2_O followed by short incubation in ddH_2_O before incubating for 15 min in Weigert’s hematoxylin [Eisenhämatoxylin A according to Weigert (Carl Roth), Eisenhämatoxylin B according to Weigert (Carl Roth); mix solution A and B 1:1]. Sections were washed in ddH_2_O and the following in H_2_O. Brilliant Crocein-acid fuchsine solution [solution A: 0.1% (w/v) Brilliant Crocein R (Chroma, Hamburg, Germany) and 0.5% (v/v) glacial acetic acid in ddH_2_O; solution B: 0.1% (w/v) acid fuchsine (Merck KGaA, Darmstadt, Germany) and 1% glacial acetic acid in ddH_2_O; mix solutions A and B 5:1] was applied for 15 min, followed by incubation in 0.5% (v/v) acetic acid, in 5% (w/v) phosphotungstic acid (Chroma, Hamburg, Germany) (20 min), and in 0.5% acetic acid again. After incubation in 100% ethanol, sections were incubated in ethanol for 1 h in a saturated solution of Saffron du Gâtinais (Chroma, Hamburg, Germany). After incubation in ethanol and xylene (AppliChem GmbH, Darmstadt, Germany), sections were mounted as described in the previous section in a mounting medium containing xylene (Entellan; Sigma-Aldrich, Taufkirchen, Germany).

### Von Kossa staining

Cryosections were washed in ddH_2_O before staining for 30 min in a 5% (w/v) silver nitrate (Carl Roth, Karlsruhe, Germany) solution. After washing in H_2_O, sections were incubated in 5% (w/v) sodium carbonate (Carl Roth, Karlsruhe, Germany) (w/v) in 9% (v/v) formaldehyde for 2 min. Before and after incubation in 5% sodium thiosulfate (w/v) (Carl Roth, Karlsruhe, Germany), sections were washed in H_2_O. Before embedding in a mounting medium containing xylene, sections were washed in ddH2O, 100% ethanol, and xylene.

### Microscopy of histochemical and immunostainings

Images were taken at 4 × magnification and then stitched using the BZ-X800 microscope and the BZ-X800 Analyzer tool from Keyence.

Immunofluorescence images were taken with an Axio Observer Z1 microscope from Zeiss. Stitched mosaic images of whole valves were recorded at 10 × magnification; detailed images were recorded at 40 × magnification using an oil immersion objective and the ApoTome device of the microscope.

### Micro-computed tomography

Preserved aortic valve tissue samples were thawed and analyzed using a micro-CT (SkyScan 1174 X-ray microtomography, Bruker, Karlsdorf, Germany). Image acquisition was performed with a rotation step of 0.2°, a pixel size of 25.5 µm, an X-ray tube voltage of 50 kVP, a current of 800 µA, and an integration time of 2200 ms; no filter was used. Generated images were reconstructed, reoriented spatially, and analyzed using purpose-built software (NRecon/DataViewer/CTAN; SkyScan, Bruker). For visualization, 3D reconstruction was performed using ImageJ. The Trainable Weka Segmentation algorithm in ImageJ performed area measurements on single micro-CT images. The 3mensio software (Pie Medical Imaging, Maastricht, The Netherlands) was used to image clinical CT data obtained before surgery.

### Ethics and permit

Patient data were processed and analyzed in an anonymized form. The clinical studies complied with Good Clinical Practice guidelines and were approved by the local ethics committee of the University Hospital Bonn, according to No. 077/14 and 078/17. The studies were performed in accordance with the Declaration of Helsinki and the International Conference on Harmonization of Good Clinical Practice, and all patients provided written informed consent.

### RNA isolation, library preparation and bulk RNA-Seq analysis

RNA isolation was carried out using the RNeasy Plus Micro Kit (Qiagen, Hilden, Germany) according to the manufacturer’s instructions. Briefly, small pieces (diameter ~ 3 mm) of the obtained valves were lysed in RLT + lysis buffer containing 1% β-mercaptoethanol using 7 mm stainless steel beads for 5 min at 50 Hz (TissueLyser LT, Qiagen, Hilden, Germany). Afterward, the supernatant was filtered through QIA shredder columns (Qiagen, Hilden, Germany) as recommended by the manufacturer and further processed with the above-mentioned kit. RNA quality was determined using a 2100 Bioanalyzer (Agilent, Santa Clara, CA, US). Only samples with an RIN > 6.5 were used for further analyses. In total, 13 bicuspid stenotic valves, 14 tricuspid stenotic valves, and 7 control valves from patients suffering from aortic insufficiency were analyzed. Libraries were prepared using the Trio RNA-seq library preparation kit (Tecan, Männedorf, Switzerland) according to the manufacturer’s recommendations. Libraries were sequenced with 25 mio paired-end reads per sample with a length of 75 bp each at EMBL (Heidelberg, Germany). Bioinformatic analyses were carried out using the European Galaxy Platform (Department of Bioinformatics, University of Freiburg, Germany [49]). For differential expression analyses, DESeq2 was used. DEGs expressed in both comparison groups, but present in less than 30% of samples, were further filtered out. The Z-scores were computed and visualized as heatmaps using the heatmap2 tool on the Galaxy Platform.

### Clinical study

The study cohort comprised 1,108 patients with severe native aortic valve stenosis who underwent TAVI at the Heart Center Bonn between January 2015 and March 2023. All patients received a comprehensive preoperative evaluation, which included transesophageal echocardiography (TEE), computed tomography (CT), and coronary angiography. Following this evaluation, all cases were discussed by the local interdisciplinary heart team. Patients were stratified based on the morphology of the aortic valve, as determined by pre-interventional TEE and CT scans, into a bicuspid aortic valve (BAV) group of 25 patients and a tricuspid aortic valve (TAV) group of 1083 patients. Pre-interventional CT scans were further analyzed using 3mensio Structural Heart software (Pie Medical Imaging, Maastricht, The Netherlands) to quantify the extent of aortic valve and iliofemoral calcification. To enable better comparison of calcification between groups, aortic valve calcification was expressed as the calcium volume of the aortic valve cusps normalized to the area of the aortic annulus. Iliofemoral calcification was measured from the femoral artery bifurcation to the aortic bifurcation. As previously reported, an adjusted threshold of 550, 300, and 50 Hounsfield units (HU) was applied to assess calcium volume scoring in patients with luminal attenuation values of 200–500 HU, < 200 HU, and > 500 HU, respectively [3].

### Statistical analysis

Statistical analyses were carried out using GraphPad Prism 10.2.3. Data are presented as means ± standard deviation (SD). Data were tested for normal distribution by the Kolmogorov–Smirnov test. Expression effects of three groups were compared using one-way analysis of variance (ANOVA) with Bonferroni’s post hoc test. *p* values < 0.05 were considered to be statistically significant. Data were normally distributed, and no outliers were detected. Statistical analyses of the clinical data were performed using SPSS version 28 (IBM Corporation, Somers, NY, USA). Continuous variables are presented as means with standard deviations if normally distributed and as medians with interquartile ranges if not. The Kolmogorov–Smirnov test was used to assess the normality of distribution for continuous variables. These variables were tested with the Student’s t test or the Mann–Whitney U test, depending on the distribution. Categorical variables are presented as absolute numbers and percentages. Differences in categorical variables were assessed using Fisher’s exact test. Statistical significance was defined as a two-tailed *p* value of ≤ 0.05. All authors vouched for the accuracy of the data and analyses.

## Results

### Gene expression analysis in AVS and bAVS

Freshly explanted aortic valves of patients with severe AVS or bAVS were collected in the operating room, and valve leaflets were either processed for histological stainings or frozen for subsequent RNA isolation and bulk RNA sequencing (Supplemental Fig. 1A). In total, 64 aortic valves (75 leaflets) from 61 different patients were used (9% women). Images of explanted AVS, bAVS, and non-calcified aortic regurgitation (AR) control valves show calcifications typical for AVS and bAVS (Supplemental Fig. 1B).

**Fig. 1:**
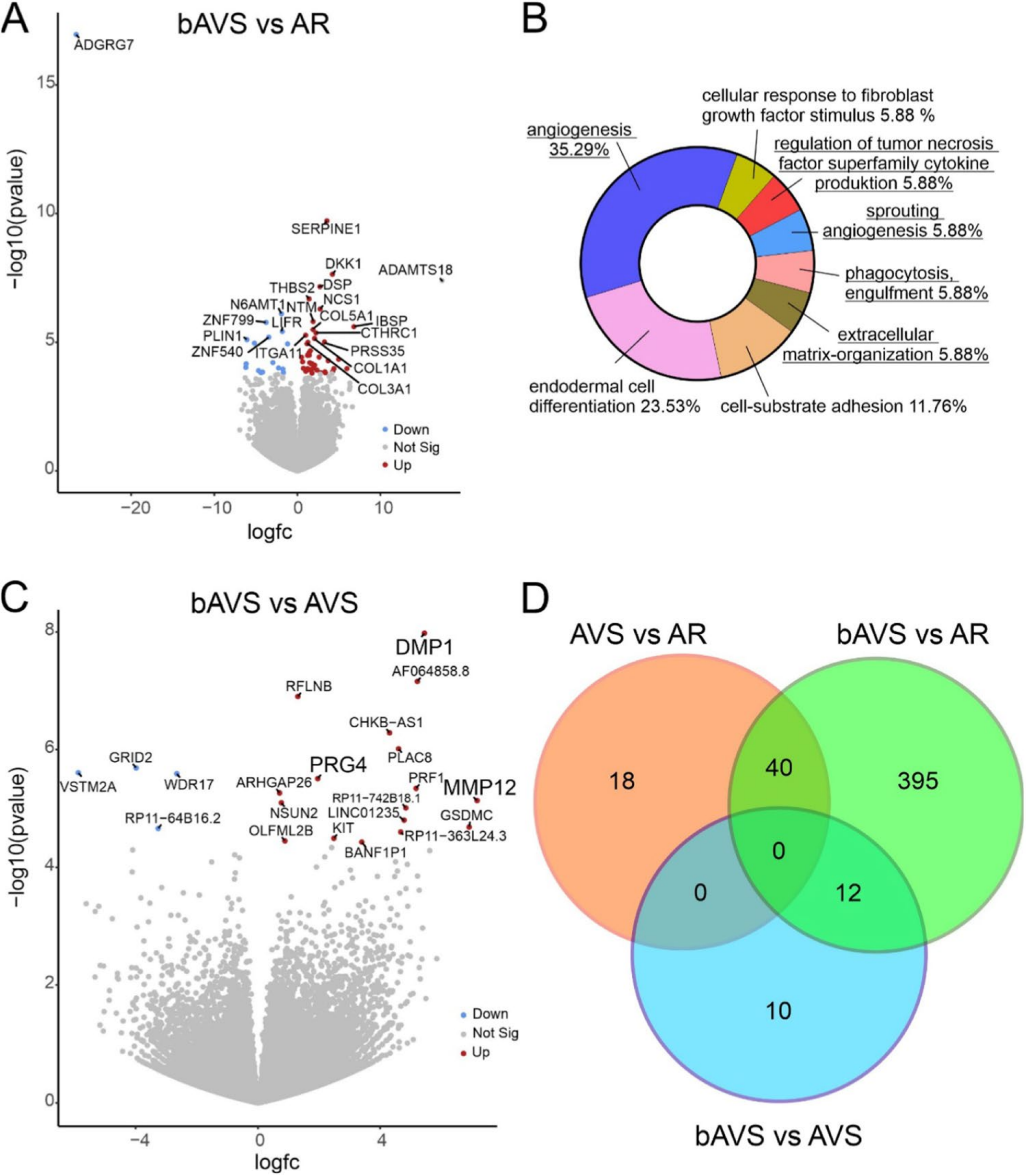
Bulk RNA-seq analysis of bAVS vs AR, and bAVS vs AVS:** A** volcano plot of the up- and downregulated genes in bAVS (*n* = 13) vs AR (*n* = 7). Genes with *p* < 0.05 and log2FC > 1 are highlighted in color. The 20 most significantly differentially expressed genes (DEGs) are labeled by name. **B** GO analysis of the upregulated genes. Categories related to angiogenesis, inflammation, extracellular matrix organization, and calcification are underlined. **C** volcano plot of the up- and downregulated genes comparing bAVS (*n* = 13) with AVS (*n* = 14). Genes with *p* < 0.05 and log2FC > 1 are color-coded (red: upregulated; blue: downregulated). The 21 most significant DEGs are labeled by name; *DMP1*, *PRG4*, and *MMP12* are highlighted. **D** Venn diagram of DEGs of the different comparisons

To explore potential differences in the pathomechanisms of AVS and bAVS, we performed in-depth bulk RNA-seq analyses and compared the gene expression profiles of AVS (*n* = 14) and bAVS (*n* = 13) with those of AR controls (*n* = 7). Interestingly, this analysis revealed only a relatively small number of differentially expressed genes (DEGs) (58 in total; 41 up- and 17 downregulated) between AVS and AR controls (Supplemental Table 2). In contrast, when comparing bAVS with AR controls, a significantly higher number of DEGs (447 in total; 298 up- and 149 downregulated) was identified (Fig. 1A; Supplemental Table 3). Gene Ontology (GO) analysis of upregulated genes in the bAVS group yielded terms such as angiogenesis/vascular development, extracellular matrix (ECM) organization, and regulation of tumor necrosis factor superfamily cytokine production, suggesting that angiogenesis, immune response, extracellular matrix remodeling, and calcification are involved in the disease process (Fig. 1B; Supplemental Table 4). Next, we compared bAVS vs. AVS to obtain more information on potentially different disease mechanisms (Fig. 1C). We found 21 (17 up-, 4 downregulated) DEGs. In particular, genes involved in the calcification process, such as dentin matrix acidic phosphoprotein 1 (*DMP1)*, proteoglycan 4 (*PRG4)*, and matrix metallopeptidase 12 (*MMP12)*, were strongly upregulated in bAVS (Supplemental Table 5). Additionally, we examined common genes that were differentially expressed in AVS and bAVS vs. AR, or were specific to either AVS or bAVS (Fig. 1D). Among the 40 jointly differentially expressed genes were integrin-binding sialoprotein (*IBSP)*, various collagens (e.g., collagen type I alpha 1 chain (*COL1A1)*, collagen type IV alpha 1 chain* (COL4A1)),* and serpine family E member 1 (*SERPINE1)*. Notably, members of the matrix and ADAM metalloprotease families, which are involved in ECM processing, along with members of the Fc gamma receptors (FCGR) and interleukin/chemokine families, which are involved in inflammation, were only upregulated in bAVS vs. AR, but not in AVS. Therefore, we focused our further analyses on angiogenesis, inflammation, ECM composition, and calcification.

### Processing of strongly calcified human aortic valves with Kawamoto’s film method

We also aimed to investigate protein expression and distribution using immunostainings. However, the standard decalcification procedure routinely used for cryosectioning calcified aortic valves often compromises the tissue sample integrity and staining quality, similar to the effects of chelating agents or acids. Because preserving the structural integrity of calcified aortic valve tissue can enhance our understanding of disease processes, we evaluated Kawamoto’s film method for this purpose**.** In this method, a cryofilm is applied to the specimen before sectioning with a tungsten carbide blade. This allows the freshly cut sections to adhere to the film, thereby potentially maintaining the structural integrity of even heavily calcified aortic valve samples. We first compared conventional cryosectioning with the Kawamoto method on calcified aortic valve samples. The hard tungsten carbide blade effectively cut through aortic valve tissue with significant calcifications, and the cryofilm prevented the calcified regions from crumbling and disrupting adjacent tissue. In contrast, conventional cryosectioning, whether or not decalcification was performed (Supplemental Fig. 2C,D) (decalcified valves *n* = 20, non-decalcified valves *n* = 20), often resulted in folding and a severe loss of structural integrity, which was also caused by displaced calcified material. By comparison, sections prepared with Kawamoto’s film method (Supplemental Fig. 2D) were well-preserved, even when processing heavily calcified human aortic valves (*n* = 58). This is particularly relevant for the endothelial cell layer, which surrounds healthy aortic valves and serves as an important barrier. Unlike the conventional method, Kawamoto sections preserved an intact CD31^+^ endothelial cell layer even in areas near heavily calcified regions in AVS and bAVS (Supplemental Fig. 2D, inset). These findings further highlight the excellent preservation of strongly calcified aortic valve tissue using Kawamoto’s film method, as even the delicate endothelial cell layer remained intact. In addition, the apotome device of the microscope was used in order to obtain sharper, high-contrast images (Supplemental Fig. 2B).

**Fig. 2:**
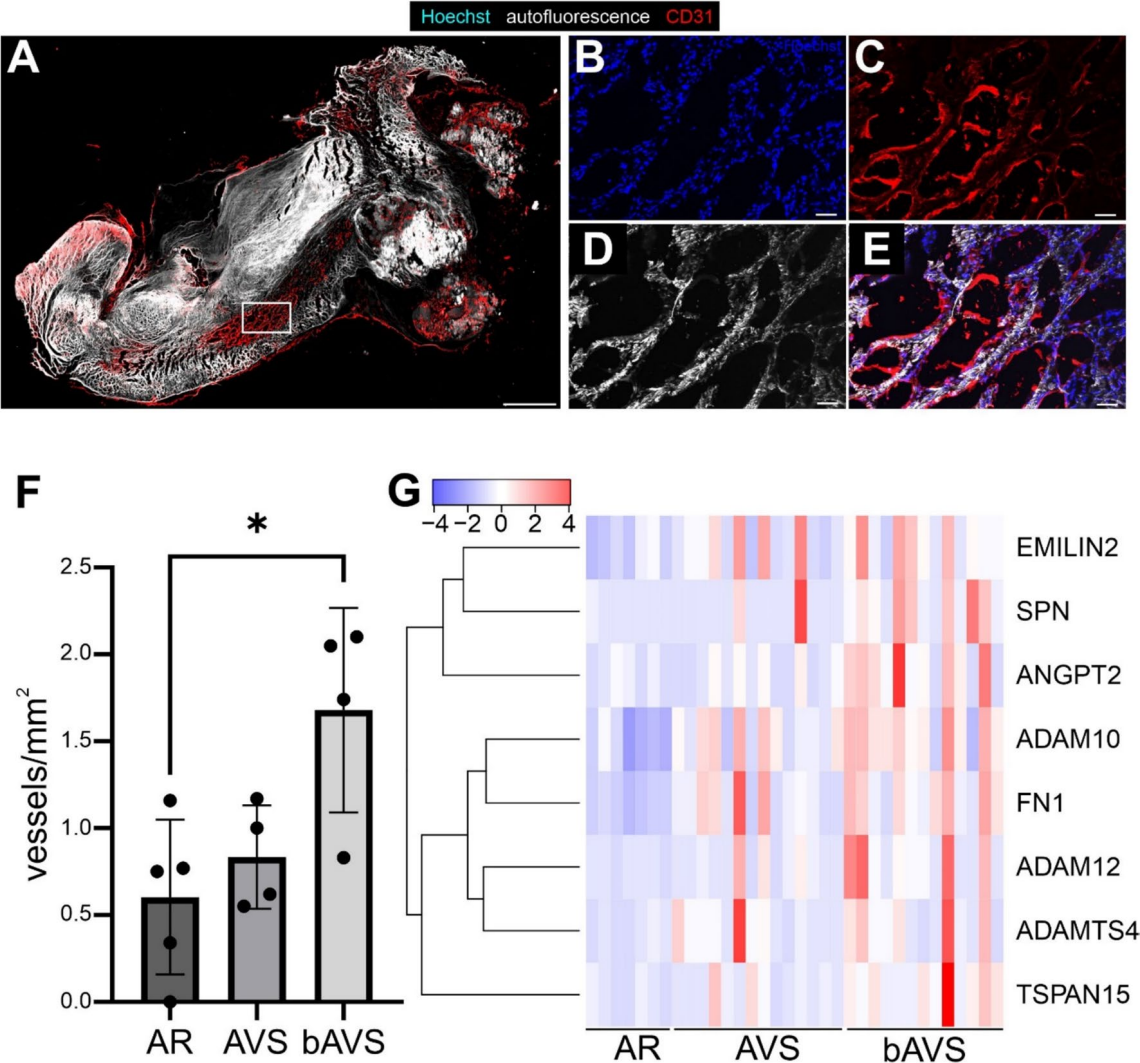
Assessment of angiogenesis in aortic valves using immunostainings and bulk RNA-seq analysis: **A–E** bAVS sample stained for CD31 (red) and nuclei (blue); magnification of the boxed area in A. **B–E** Scale bar = 1000 µm for overview image, 50 µm for inset. **F** Quantitation of CD31^+^ vessels. *p < 0.05; analyzed by one-way ANOVA. **G** Heatmap of bulk RNA-seq data (AR: *n* = 7, AVS: *n* = 14, bAVS: *n* = 13)

### Angiogenesis, inflammation, and endothelial barrier function in AVS and bAVS

Since Kawamoto’s film method greatly improved the histomorphological preservation of aortic valve samples, we next investigated angiogenesis by staining for CD31. This was also prompted by earlier studies suggesting that neovascularization is altered in bAVS compared with AVS [34]. Immunohistochemical analysis demonstrated numerous endothelial cells in bAVS samples (Fig. 2A–E), indicating angiogenesis. In fact, quantitation of CD31^+^ vessels revealed a significantly higher number in bAVS (*n* = 4) than in AR controls (*n* = 5; Fig. 2F). Although an increase was also observed when comparing bAVS with AVS, this difference did not reach statistical significance (*p* = 0.0621). To further explore the mechanism underlying increased angiogenesis, we examined our bulk RNA-seq data at the gene level, since GO analysis had already indicated increased angiogenesis. As illustrated in the heatmap (Fig. 2G), bAVS samples showed strong upregulation of genes involved in angiogenesis, including angiopoietin 2 (*ANGPT2)*, emilin 2 (*EMILIN2)*, and fibronectin (*FN1).* In contrast, expression of these angiogenic genes was highly variable across AVS samples, with no clear overall upregulation of angiogenesis (Fig. 2G), consistent with our immunostaining results.

**Fig. 3:**
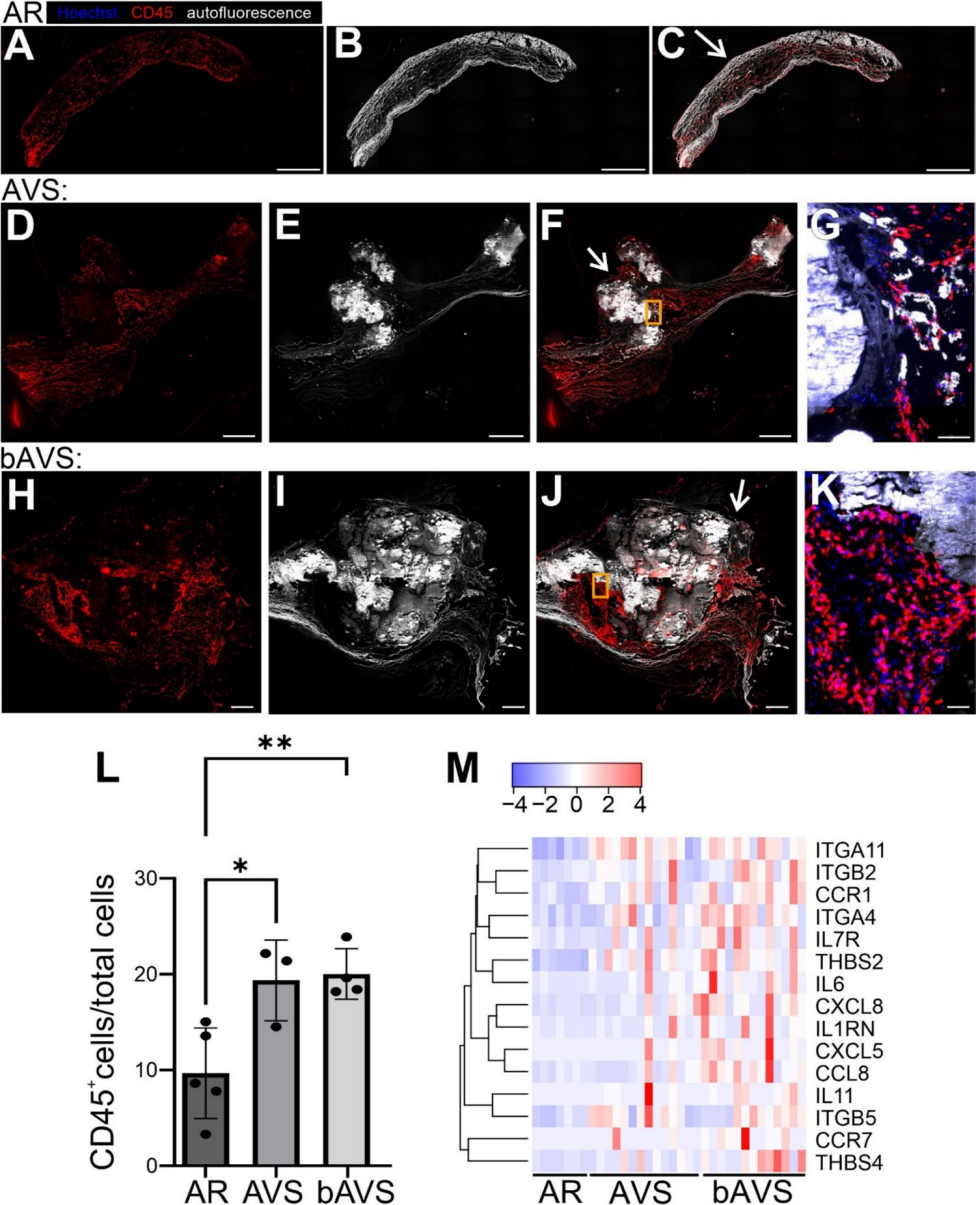
Assessment of immune cell infiltration in calcified aortic valves using immunostainings and bulk RNA-seq analysis: CD45 staining (red) in non-calcified ARs (**A–C**), AVS (**D–G**), and bAVS (**H–K**); autofluorescence is shown in white. Arrows mark the aortic side of the valves. Orange boxes in overview images mark the locations of the insets shown in G for AVS and in K for bAVS. Scale bar = 1000 µm; scale bar for insets = 100 µm. **L** Quantitation of CD45^+^ cells in aortic valves. **M** Heatmap of chemokines and immune cell genes in AVS and bAVS vs. AR controls

Because angiogenesis is associated with immune cell infiltration and inflammation [47], both of which are thought to contribute to aortic valve stenosis [50, 52], we investigated these processes using immunostaining and bulk RNA-seq. Immune cell infiltration was assessed by staining with the pan-hematopoietic marker CD45. In AR samples (*n* = 5; Fig. 3A–C), only a moderate number of CD45 + cells were observed. In contrast, both AVS (*n* = 3) and bAVS samples (*n* = 4) exhibited a marked increase in CD45^+^ cells, primarily located near calcified areas (Fig. 3D–K). Quantitative analysis confirmed a significant increase in CD45^+^ cells in AVS (*n* = 3) and in bAVS (*n* = 4) compared to AR controls (*n* = 5; Fig. 3L). RNA-seq analysis revealed a significant upregulation of inflammatory genes including interleukins, namely interleukin 1 receptor antagonist (*IL1RN),* interleukin 7 receptor* (IL7R*), interleukin 6* (IL6),* interleukin 11* (IL11)*, chemokines (C–C chemokine motif ligand 8 (*CCL8),* C-X-C motif chemokine ligand 5 (*CXCL5*), C–X–C motif chemokine ligand 8 *(CXCL8),* C–C chemokine motif receptor 1 *(CCR1),* C–C chemokine motif receptor 7* (CCR7)*) and integrins (integrin subunit alpha 4 (*ITGA4),* integrin subunit alpha 4 (*ITGA11),* integrin subunit beta 2* (ITGB2),* integrin subunit beta 5* (ITGB5*)) along with their respective receptors in calcified bAVS (*n* = 13) compared to non-calcified AR controls (*n* = 7; Fig. 3M). Interestingly, earlier studies have also reported increased expression of chemokines, such as C–C chemokine motif ligand 21 (*CCL21/CCR7*), elevated IL6 levels in the serum of AVS patients, and higher *CXCL8* levels in aortic disease [14, 23, 51]. Although these genes were also upregulated in AVS (*n* = 14) compared to AR controls, the increase was less pronounced. Overall, these findings indicate strong immune cell infiltration and heightened inflammation in bAVS, highlighting a potentially crucial role for these processes in valve calcification, as suggested in the literature [50, 52].

Due to increased immune cell infiltration and inflammation, we also evaluated endothelial barrier function by co-staining for CD31 and albumin. As expected, albumin was visible on the endothelial cell layer in AR (*n* = 6), indicating an intact barrier that prevents albumin from passing transendothelially (Supplemental Fig. 3A–C). In clear contrast, in calcified AVS (*n* = 9) and bAVS (*n* = 13) samples, albumin was detected within the valve tissues beneath the endothelial cell layer, indicating disruption of the endothelial barrier function (Supplemental Fig. 3D–I), as previously suggested for AVS [30].

### Extracellular matrix and calcifications in AVS and bAVS

**Fig. 4:**
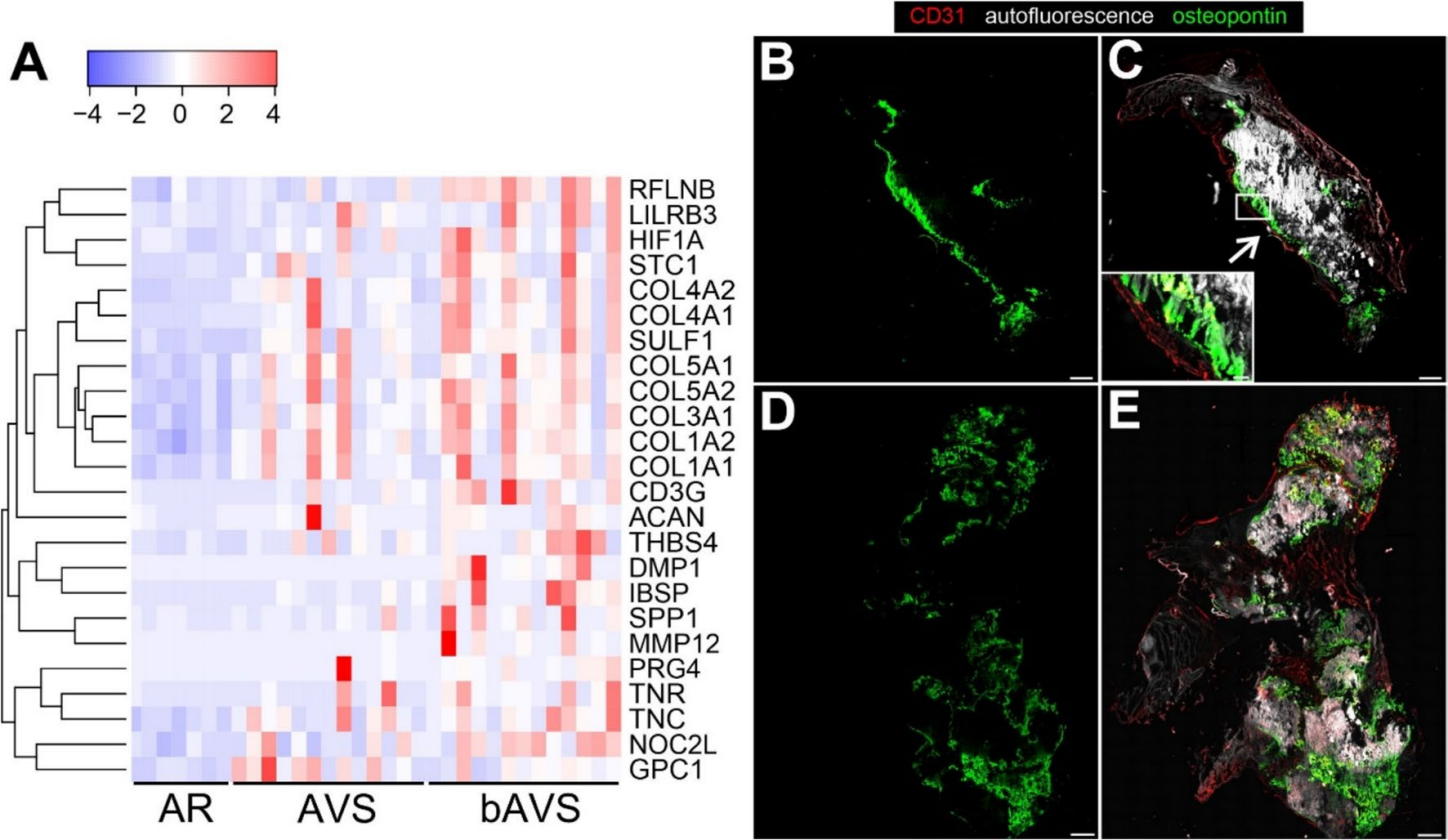
RNA-seq analysis of calcification markers, and assessment of osteopontin expression in calcified aortic valves and controls using immunostainings: **A** Heatmap showing ECM-related gene expression in AR, AVS, and bAVS samples; SPP1 encodes osteopontin. **B, C** Staining for osteopontin (green), CD31 (red), and autofluorescence (white) labeling endothelial cells and calcifications in an AVS sample. The white arrow in C points to the aortic side of the valve, the boxed area labels the section shown at higher magnification in the inset. **D, E** Stainings as in **B, C** in a bAVS sample. Scale bar = 500 µm; scale bar for inset = 50 µm

Given the importance of fibrosis and calcification in aortic valve stenosis, we conducted a detailed analysis of these processes. We found that many genes significantly upregulated in bAVS (*n* = 13) compared to AR controls (*n* = 7) are involved in calcification and ECM biology. These include genes related to mineralization, bone formation, and calcium metabolism, such as the proteoglycan aggrecan (*ACAN*), stanniocalcin (*STC1*), *DMP1,* hypoxia inducible factor 1 subunit alpha (*HIF1A),* and also secreted phosphoprotein 1 (*SPP1)*, which encodes osteopontin (Fig. 4A*)*. Although the upregulation of these genes was less pronounced in the AVS group (*n* = 14), genes associated with calcification, such as *IBSP* and various collagens (e.g., *COL1A1*, collagen type IV alpha 2 chain (*COL4A2*)), were also differentially expressed in AVS vs. AR (Fig. 4A), which aligns with the presence of calcifications in these valves. Notably, as noted above, genes encoding proteins known to play important roles in ECM remodeling and calcification, namely *DMP1, MMP12,* and *PRG4*, were significantly more highly expressed in bAVS than in AVS, suggesting a potentially important pathophysiological role in this disease. Osteopontin is a secreted phosphoprotein expressed in osteoblasts and serves as a marker for bone formation and bone-like structures [33]**.** To further investigate the localization and extent of calcification**,** we performed osteopontin staining in AVS (*n* = 6) and bAVS (*n* = 4) samples. Notably, in both groups, osteopontin expression was primarily observed in areas surrounding acellular calcifications, specifically in the outer layers of the calcium deposits (Fig. 4B–E). We also tested various methods to visualize calcifications in aortic valves, and found that background fluorescence in Kawamoto’s film sections provided the best results (Fig. 4C).

**Fig. 5:**
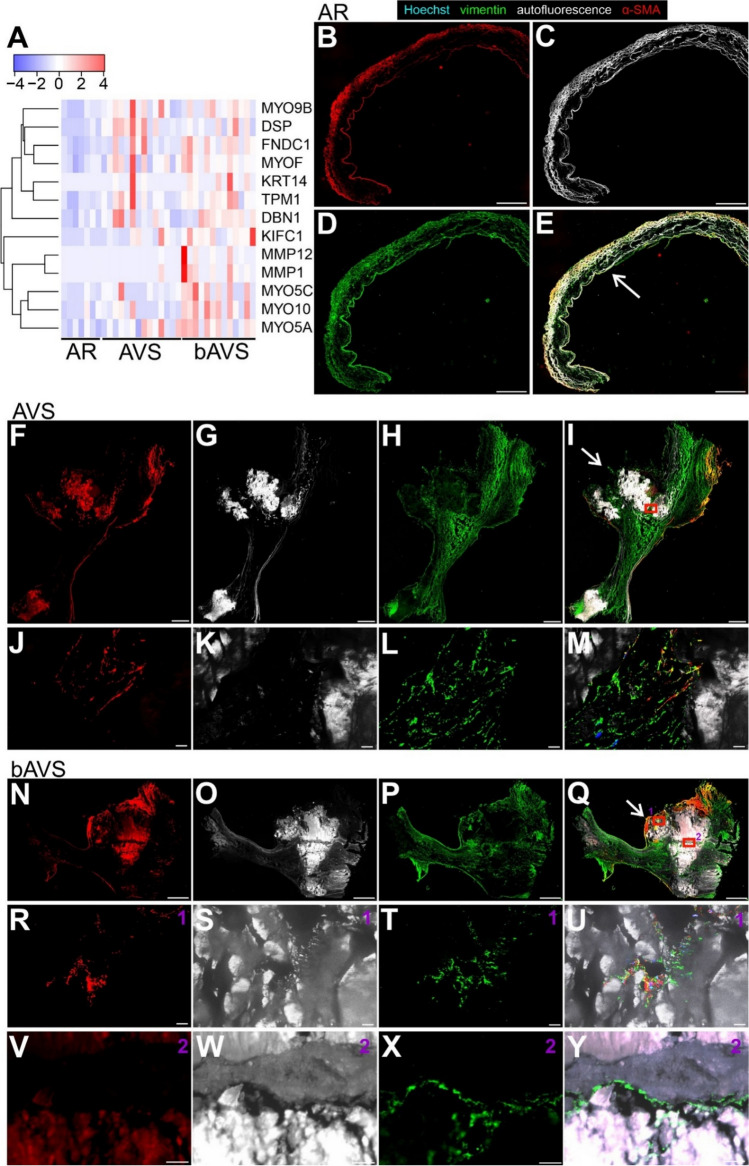
RNA-seq analysis of cellular structure markers, and α-SMA and vimentin expression in calcified aortic valves and controls using immunostainings: **A** Heatmap shows expression of structural proteins and cytoskeletal markers in AVS and bAVS compared to ARs. Staining for vimentin (green) and α-SMA (red) in non-calcified ARs (**B-E**), AVS (**F-M**), and bAVS (**N-Y**), autofluorescence is shown in white, and nuclei are stained with Hoechst. White arrows in the merged images mark the aortic side of the valves. Red boxes with numbers in overview images identify areas selected for insets and their corresponding insets. Scale bar = 500 µm for overview images, 50 µm for insets

A key cellular component of aortic valves, besides VECs, is VICs. VICs are a heterogeneous group of cells that display characteristics of fibroblasts, myofibroblasts, and smooth muscle-like cells [13]. When analyzing the DEGs and their associated expression patterns, we observed upregulation of many genes involved in cell structure, cell adhesion, and cytoskeletal organization in bAVS (*n* = 13). The most strongly upregulated genes included several myosins [myosin VA (*MYO5A),* myosin X* (MYO10),* myosin IX B* (MYO9B),* myoferlin* (MYOF)*], *MMP12*, matrix metallopeptidase 1 (*MMP1),* and desmoplakin (*DSP*), consistent with the prominent calcifications observed in these valves (Fig. 5A). Although these genes were also upregulated in AVS (*n* = 14), the increase was less pronounced compared to AR (*n* = 7; Fig. 5A), with only *DSP* showing significant upregulation in AVS relative to AR.

Next, we performed immunostaining on Kawamoto-processed aortic valves for vimentin, a common VIC marker, and α-SMA. In non-calcified aortic valves, α-SMA is typically localized to the ventricular side, but earlier studies have reported abnormal localization in AVS, with α-SMA expressed on both sides [26]. Accordingly, we found that in AR (*n* = 6), α-SMA was mainly confined to the ventricular side (Fig. 5B–E). In contrast, in both AVS (*n* = 7) and bAVS (*n* = 7), α-SMA was detected on both sides of the valve (Fig. 5F–Y) and in regions adjacent to calcifications. We also examined the distribution of VICs, focusing on calcifications and their surrounding regions. In non-calcified AR, vimentin was widely distributed throughout the valve, with more prominent expression on the ventricular side. In both AVS and bAVS, vimentin expression was confined to soft tissue, with no vimentin^+^ cells observed in heavily calcified regions. However, vimentin^+^ cells were found between areas of early, non-compact calcifications (Fig. 5J–M, R–Y) and also between regions of dense calcified material (Fig. 5H, P). Evidence suggests that in AVS, VECs of the aortic valve undergo EndoMT during calcification [29]. However, our co-staining experiments for α-SMA or vimentin with CD31 (data not shown) showed no evidence of EndoMT.

### Correlating CT scan, histomorphological, and clinical data in AVS and bAVS patients

The excellent preservation of Kawamoto-processed AVS and bAVS samples allowed us to compare the histomorphological features of heavily calcified aortic valves in patients both before and after surgical removal. To this end, we correlated clinical CT scans with micro-CT, histological, and immunostaining data from the same-valve leaflets. Micro-CT analysis enabled a direct comparison of the clinical severity of valve stenosis with the degree of leaflet calcification in patients with severe aortic valve stenosis.

**Fig. 6:**
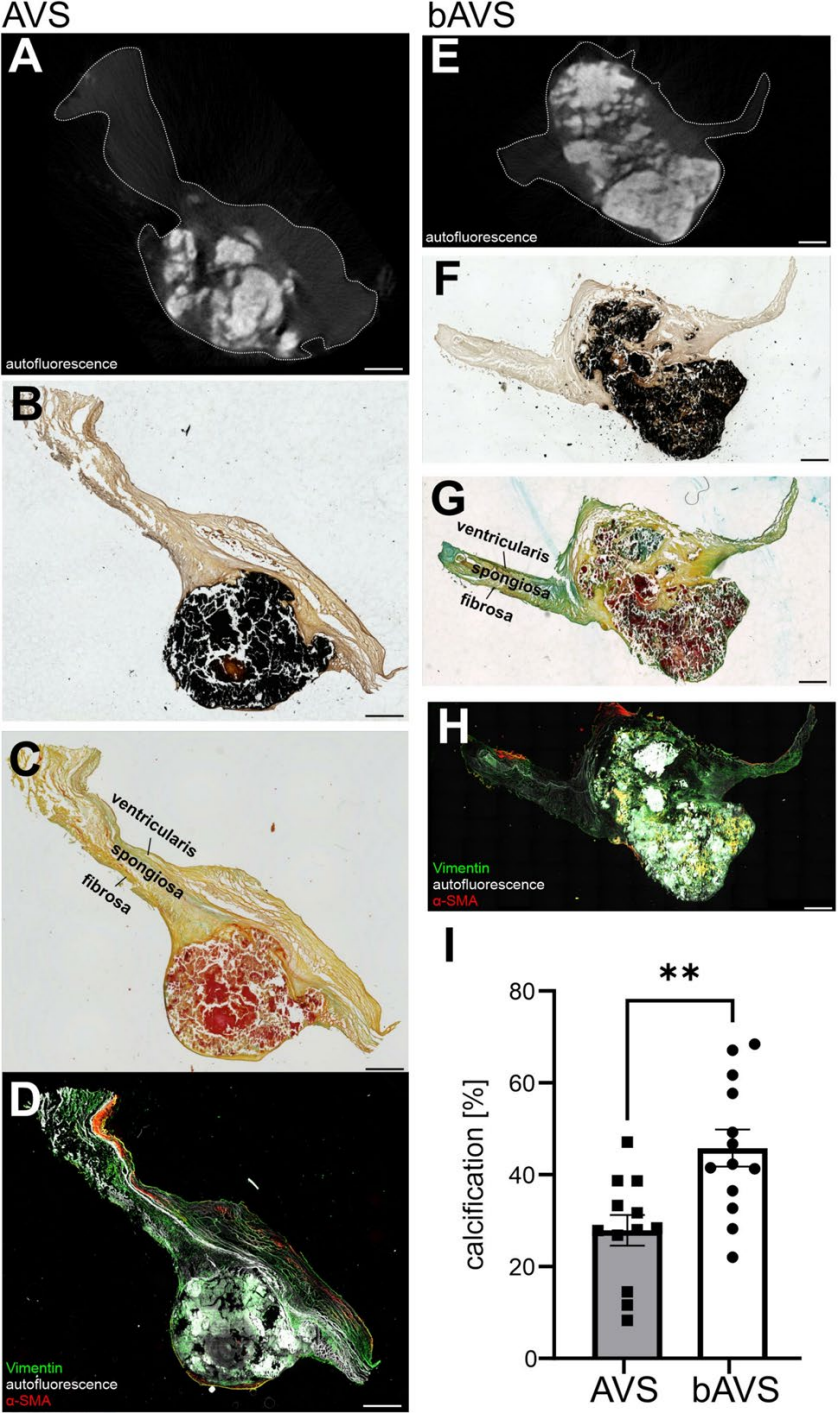
Micro-CT and histomorphological characterization of calcifications in aortic valves: Micro-CT and histomorphological analysis of a calcified AVS (**A–D**) and bAVS (**E–H**). **A, E** Quantitative assessment of the calcified area percentage in valve slices, tissue borders are marked with dashed lines. **B, F** Detection of calcifications using von Kossa stainings. **C, G** Movat-Pentachrome stainings in adjacent sections of the von Kossa stainings. **D, H** Calcifications visualized in the autofluorescence channel (white). **I** Quantitation of calcifications in AVS (*n* = 12) and bAVS (*n* = 13) samples using von Kossa staining. Scale bar = 1000 µm

Micro-CT data highlighted well-preserved morphological features in both AVS (Supplemental Fig. 4A, B) and bAVS (Supplemental Fig. 4E, F). Notably, the micro-CT images of the AVS (Supplemental Fig. 4A) and bAVS (Supplemental Fig. 4E) closely matched the corresponding clinical CT scans (Supplemental Fig. 4B, F, blue-colored leaflet) acquired before surgery. We further correlated micro-CT findings from explanted valve leaflets with histomorphological data and assessed the extent and distribution of the calcifications [41]. In addition, 3D reconstruction of micro-CT data enabled visualization and quantitation of the calcification volume in AVS (Supplemental Fig. 4C) and bAVS (Supplemental Fig. 4G). The valve leaflets are shown from a top view in the clinical CT scans, with the same leaflets examined by micro-CT and immunohistology highlighted in blue for AVS (Supplemental Fig. 4D) and bAVS (Supplemental Fig. 4H). Calcification in the micro-CT slice of the AVS (Fig. 6A) appears as a bright white area with a faint signal (dashed line) delineating the surrounding soft tissue. Quantitative analysis revealed that calcification occupied 32.6% of the area and 14.4% of the volume within the leaflet (Fig. 6A–D). To correlate micro-CT findings with histological features, the leaflet was cryosectioned along the same axis and further analyzed using von Kossa (Fig. 6B) and Movat-Pentachrome (Fig. 6C) stainings. Von Kossa staining (Fig. 6B) demonstrated extensive calcifications on the aortic side of the leaflet, which was corroborated by Movat-Pentachrome staining. According to the literature [2], the different colors in Movat-Pentachrome staining allow identification of the ventricular and aortic sides of the aortic valve and distinguish between stages of calcification, as mineralized cartilage or bone tissue stains dark blue, whereas newly formed osteoid-like structures appear dark red. In the analyzed AVS, only a single stage of calcification was detected, as the valve leaflet stained uniformly red (Fig. 6C). We also performed immunostainings for vimentin and α-SMA on consecutive sections of the leaflets (Fig. 6D) and found that vimentin was widely expressed throughout the valve, except in areas of dense calcifications. Again, aberrant ɑ-SMA expression was observed on the aortic side of this AVS (Fig. 6D). In contrast, calcification in the bAVS (Fig. 6E–H), as measured by micro-CT (Fig. 6E), accounted for 58.7% of the total valve leaflet area and 42.1% of the volume. Von Kossa staining (Fig. 6F) highlighted extensive calcifications on both sides of the aortic valve, while Movat-Pentachrome (Fig. 6G) staining showed different stages of calcification, a mineralized stage (dark blue) on the ventricular side, and a more recently formed stage (red) on the aortic side. To quantify calcified areas in valve leaflets, they were normalized to the total leaflet area in von Kossa stainings. Our analysis yielded a significantly higher degree (1.7-fold) of calcification in bAVS (46.6 ± 15%, *n* = 13) compared to AVS (27.9 ± 11,6%, *n* = 12, *p* = 0.0025, Fig. 6I). Thus, gene expression, micro-CT, and histological analyses indicate more extensive calcifications in bAVS compared to AVS.

**Fig. 7:**
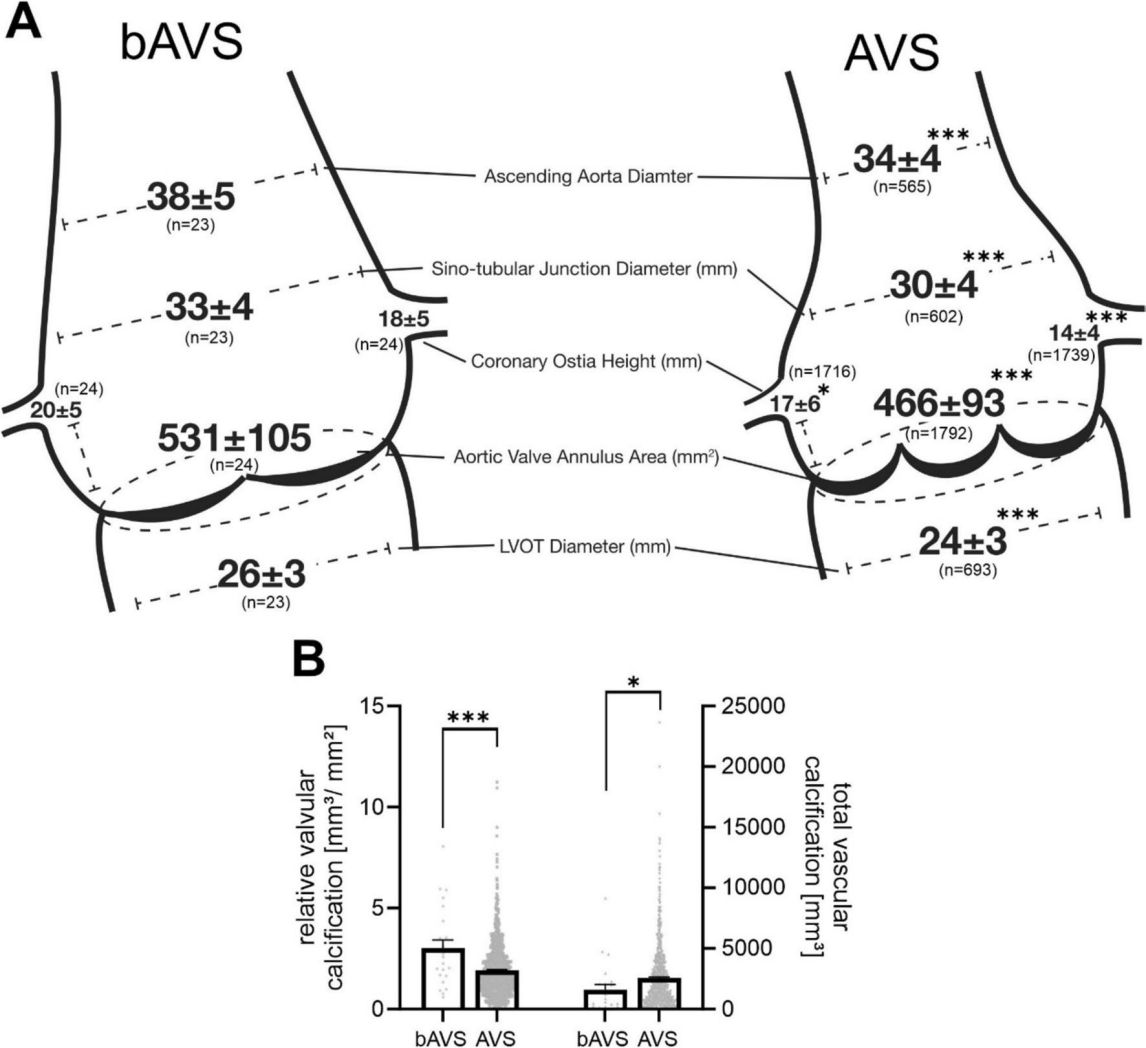
Retrospective analysis of clinical aortic valve parameters in AVS and bAVS patients before TAVI (*n* = 1108). **A** CT-based diameters of the aortic and aortic valve annulus areas in bAVS (left) and AVS (right) patients. **B** Statistics of CT-based assessment of relative valvular and total vascular (iliac arteries) calcification

Given the significant differences in calcification levels between AVS and bAVS, we conducted a retrospective analysis of 1108 patients with severe AVS or bAVS undergoing TAVI. Both groups showed similar baseline clinical characteristics (Supplemental Table 6) and had comparable mean ages. However, patients with bAVS had slightly lower body mass index (BMI), EuroSCORE II, STS-PROM, and left-ventricular ejection fraction (EF) than those with AVS. Additionally, the prevalence of arterial hypertension was lower in the bAVS cohort (Supplemental Table 6). A detailed analysis of pre-interventional CT scans showed, consistent with earlier studies, that bAVS patients had significantly larger anatomical dimensions across all measured parameters (Fig. 7A). Importantly, the total valvular calcification burden was significantly higher in bAVS patients compared to AVS patients. Notably, the relative valvular calcification (defined as total calcium load/total valve area) was also significantly higher (1.6-fold) in bAVS patients, despite their lower levels of iliac artery calcification, a marker of systemic vascular calcification (Fig. 7B). These findings align with our histological data, which show that bAVS is characterized by markedly increased localized valvular calcification, suggesting distinct pathophysiological mechanisms.

## Discussion

In this study, we collected unselected AVS, bAVS, and non-calcified AR as controls directly in the operating room and processed and analyzed the samples using a multi-technique approach. The clinical presentations and pathological features of these two forms of aortic valve stenosis differ markedly, suggesting distinct pathophysiology, even though clinical outcomes are similar. In fact, among patients undergoing surgical valve replacement, we found at the molecular level that bAVS is characterized by significantly greater angiogenesis, inflammation, and calcification than AVS. Notably, in a large, independent cohort of age-matched AVS and bAVS patients undergoing TAVI at the Bonn Heart Center, the bAVS group exhibited more localized valvular and aortic pathology and significantly greater valve calcification, whereas the AVS group showed a more systemic vascular disease pattern, including calcification. Thus, these data illustrate that AVS correlates with atherosclerosis-related diseases, such as coronary artery disease [12, 22], whereas bAVS is a distinct disease entity.

A major technical limitation of conventional decalcification and sectioning techniques for calcified aortic valves is that they often compromise tissue integrity and antigenicity, thereby hindering immunohistochemical and molecular diagnostic procedures, such as fluorescence in situ hybridization (FISH) or DNA- and RNA-based assays. We have addressed this issue by establishing Kawamoto’s film method, originally developed for bone tissue [24]. The excellent preservation of such heavily calcified aortic valve tissue was demonstrated by comparing pre-surgery CT scans of AVS and bAVS patients with micro-CT and 3D reconstructions of the same-valve leaflets processed using the Kawamoto method. In addition, this approach enabled us to combine immunohistochemical analysis with in-depth bulk RNA sequencing in the same specimen, compare the results with micro-CT analysis, and conduct a retrospective clinical study of a large patient cohort with severe aortic valve stenosis to gain deeper insight into the pathomechanism and the common and uncommon features underlying AVS and bAVS.

We found that AVS and bAVS retained an intact endothelial cell layer despite extensive calcifications, which may explain the low rate of thromboembolic complications in these patients. Endothelial barrier dysfunction, as shown by subendothelial albumin accumulation, was common in both types of aortic stenosis. Additionally, α-SMA, a general marker for VICs, was localized to the ventricular side of non-calcified aortic valves, whereas in calcified AVS and bAVS, it was also expressed on the aortic side, consistent with earlier reports [27, 45]. In both AVS and bAVS, genes involved in calcification, including *IBSP*, *SPP1*, and various collagens, were upregulated, consistent with previous studies linking these genes to aortic disease. This was also observed for *SERPINE1*, which is known to be differentially regulated in many cardiovascular diseases [42].

Beyond these common features of AVS and bAVS, we did not expect major differences, given the advanced disease stage and earlier reports documenting very high similarity in gene expression [16]. However, our RNA-seq analysis comparing bAVS with AR and AVS clearly identified differentially expressed genes associated with vascular development, immune response, ECM, and calcification. In fact, neoangiogenesis was significantly increased in bAVS, as reported earlier [34], and this finding was consistent with the observed upregulation of angiocrine and extracellular matrix genes involved in this process. Although the mechanisms driving angiogenesis in bAVS remain unclear, our data show a strong inflammatory signature in bAVS, evidenced by upregulation of interleukin (e.g., *IL6*) and chemokine genes, as well as immune cell infiltration. Given the well-established mechanistic link between inflammation and angiogenesis [47] and their mutual reinforcement in cancer biology, our findings suggest that a similar mechanism may also be present in bAVS pathology. Although earlier work suggests a mechanistic link between inflammation and aortic valve calcification [28, 50, 52], the precise role of angiogenesis remains unclear, despite circumstantial evidence from experimental studies [32, 53]. Therefore, analyzing bAVS specimens from earlier disease stages and conducting in vitro studies could provide novel insight into the interconnection among these three processes, thereby improving our mechanistic understanding of the pathophysiology. Interestingly, Houesseau et al. [20] observed increased *IL6*-related inflammation in early onset severe calcified aortic valve stenosis. Likewise, we found that *IL6* expression was upregulated in bAVS vs. AR but not in AVS vs. AR. Whether *IL6* could serve as a marker for early onset bAVS remains to be determined, as our patient cohorts had advanced disease. Likewise, *CXCL5*, which was upregulated only when comparing bAVS with AR, was associated with disease severity in the study by Houesseau et al. [20].

The most striking difference between AVS and bAVS was the expression of genes involved in calcification and the degree of calcification in two different patient cohorts, despite similar clinical stages of stenosis and age. Bulk RNA-seq revealed that the differential expression of genes involved in calcification and ECM remodeling was much more pronounced in bAVS vs AR than in AVS vs AR. In fact, *COL6A3*, *DMP1*, *MMP12*, *PRG4*, and *MMP1*, all genes related to ECM remodeling and calcification, were upregulated only in bAVS vs AR. These findings were underscored by assessing the degree of calcification in AVS and bAVS using histological and CT-based quantification in surgically removed specimens and in 1108 planned TAVI patients. Notably, calcification was 1.7-fold higher in bAVS than in AVS by von Kossa staining and 1.6-fold higher by CT analysis, indicating a high degree of overlap across patient cohorts and quantification methods; this also aligned with the previously reported 1.4-fold difference using CT-based analysis [15]. Our micro-CT analysis, combined with pentachrome staining, suggested different stages of calcification in bAVS vs AVS, but this would need to be investigated in a larger number of valves. Given the pronounced differences in calcification levels between bAVS and AVS, we next investigated whether genes known to be involved in ECM remodeling and calcification were significantly upregulated in bAVS compared to AVS, identifying *MMP12*, *DMP1*, and *PRG4*. Consistent with our results, Zamani et al. [55] also found *PRG4* upregulated in bAVS and potentially correlated with the severity of calcified aortic valve stenosis [55]. Likewise, *DMP1* has been shown to promote a pro-osteogenic response in VICs isolated from AVS via MAPK signaling and to contribute to cardiac valve calcification [7]; however, the more pronounced upregulation of *DMP1* expression in bAVS than in AVS observed herein is novel. Similarly, *MMP12* became a gene of interest, because it was found to induce a pro-osteogenic response in VICs by activating p38 MAPK-mediated LRP-6 and β-catenin signaling pathways [9] or by degrading elastin [10], and to be upregulated in both calcified bAVS and AVS [16], although more prominent expression in bAVS has not been reported previously. Thus, our findings suggest that these genes could play an important role in the onset and progression of bAVS, and future knockout and overexpression studies in vitro and in vivo could provide more mechanistic insights.

Our study also has some limitations: We used AR valves as non-calcified controls, which are known to exhibit altered ECM composition, potentially leading to an underestimation of expression differences between bAVS and AVS compared with healthy valves. That being said, the identified DEGs related to ECM remodeling meet an even higher stringency and should, therefore, be more robust and significant. Moreover, we have tried to combine RNA-seq with immunostaining from the same specimen whenever possible, and therefore, the n’s are not large. However, the overlap between gene and protein expression underscores the validity of the results. Furthermore, differences in age, risk factors, and medication between the surgically resected and TAVI patient cohorts make it unclear whether our molecular findings can be extrapolated to the TAVI group. However, as noted above, calcification levels in bAVS are nearly identical between the two cohorts despite different quantification methods.

Although bAVS is the most common congenital heart disorder [46], its pathophysiology remains poorly understood. It is thought to be linked to early embryonic developmental issues [31], such as endothelial-to-mesenchymal transition [1], as well as to acquired factors. Notably, recent genome-wide association studies (GWAS) have identified several single-nucleotide polymorphisms in bAVS patients [21], paving the way for further research on affected signaling pathways. We found that differences in inflammation, angiogenesis, tissue calcification, and associated gene expression profiles distinguish end-stage bAVS from AVS and, clinically, correspond to localized valvular and aortic pathology versus diffuse vascular disease. These processes are likely contributing to disease development and progression and should be explored in further mechanistic studies using animal models.

Since individuals with a bicuspid aortic valve are asymptomatic early in life but face a higher risk of developing bAVS and/or an ascending aortic aneurysm, as confirmed by our TAVI cohort, early identification and evaluation at a relatively young age are crucial. Identifying the mechanisms that drive disease development is essential to improving both prevention and treatment strategies. Given that screening for bAVS is feasible and that it has an earlier onset than other aortic valve diseases, with more pronounced calcification, increased angiogenesis, and heightened activation of valvular interstitial cells, these features highlight an opportunity for earlier risk stratification and intervention, assuming these processes are present in the early stages of the disease. Implementing preventive or disease-modifying therapies early may help reduce disease progression and complications and improve long-term outcomes for this particularly vulnerable patient population.

## Supplementary Information

Below is the link to the electronic supplementary material.

Supplementary file1 (XLSX 11 KB)

Supplementary file2 (XLSX 12 KB)

Supplementary file3 (XLSX 38 KB)

Supplementary file4 (XLSX 12 KB)

Supplementary file5 (XLSX 10 KB)

Supplementary file6 (XLSX 11 KB)

Supplementary file7 (DOCX 1414 KB)

Supplementary file8 (MP4 2.81 MB)

Supplementary file9 (MP4 11.8 MB)

Supplementary file10 (MP4 5.94 MB)

Supplementary file11 (MP4 28.5 MB)

## Data Availability

All data related to this study are included in the paper or supplementary materials. All sequencing data sets mentioned in this manuscript are deposited in the Short Read Archive at the National Center for Biotechnology Information under the BioProject IDs PRJNA1251826 and PRJNA1347379. Additional data supporting the findings are available from the corresponding author upon request. Source data are provided with this paper.
