# Supplementary material for: Bicuspid aortic valve stenosis is characterized by increased angiogenesis, inflammation, and a higher valvular-to-systemic calcification ratio than tricuspid aortic valve stenosis
Source: Basic Res Cardiol. 2026 Jun 27;121(4):801–16. doi: 10.1007/s00395-026-01191-8 (PMC13372849; doi:10.1007/s00395-026-01191-8)
Supplement: Supplementary file 7 — Supplementary file7 (DOCX 1414 KB) [file 395_2026_1191_MOESM7_ESM.docx]

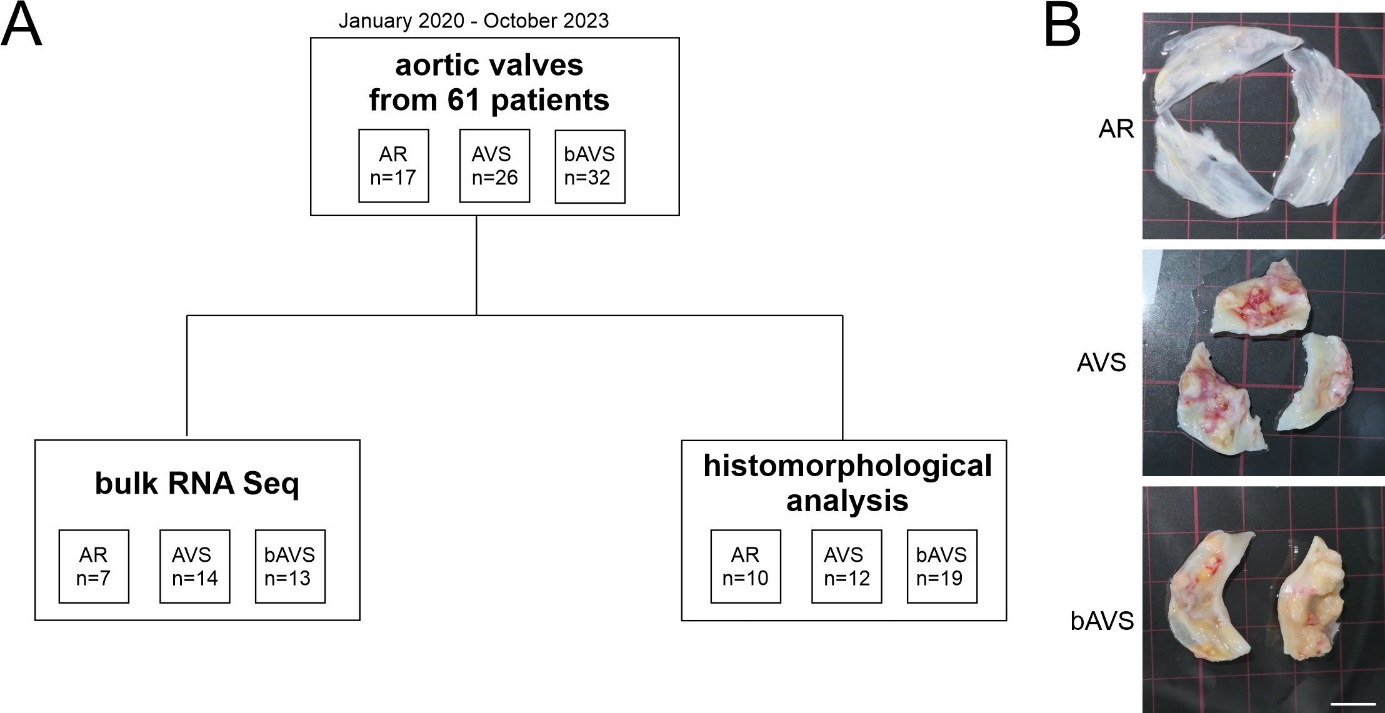


***Supplemental Figure 1: Overview of the study design and images of aortic valve leaflets of AVS, bAVS, and AR:*** ***A****. Scheme listing the number of collected valves and the analyses performed; from 14 patients, both bulk RNA Seq and histomorphological analyses were performed using different valve leaflets.* ***B****. Macroscopic images of valves from patients with tricuspid (AVS) or bicuspid (bAVS) aortic valve stenosis or aortic regurgitation (AR). Scale bar = 1 cm.*


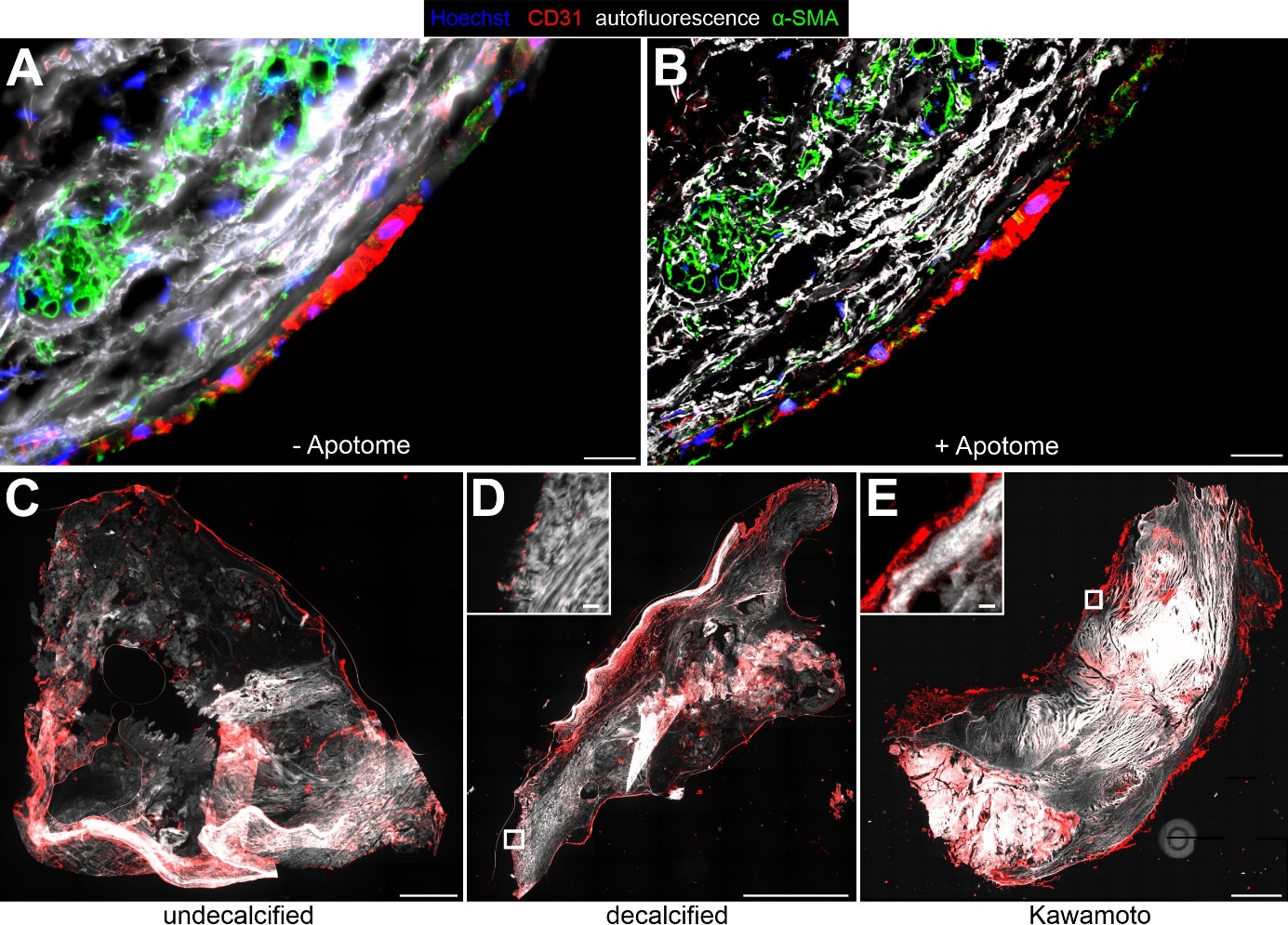


***Supplemental Figure 2: Imaging of immunostained aortic valve sections****:* ***A,B:****Images were acquired using a fluorescence microscope equipped with an oil immersion objective and without (****A****) or with an ApoTome device (****B****). Nuclei were stained with Hoechst (blue), endothelial cells for CD31 (red), and interstitial cells for α-SMA (green), respectively. Autofluorescence is shown in white. Scale bar = 20 µm.* ***C-E:*** *Cryosections of conventionally processed undecalcified (****C****), decalcified (****D****), or using Kawamoto’s film method for aortic valves (****E****). Staining for CD31 (red) and autofluorescence (white) is shown. Boxed areas in* ***D,*** ***E*** *mark the regions displayed in the insets. Scale bar = 1000 µm, scale bar for insets = 20 µm.*


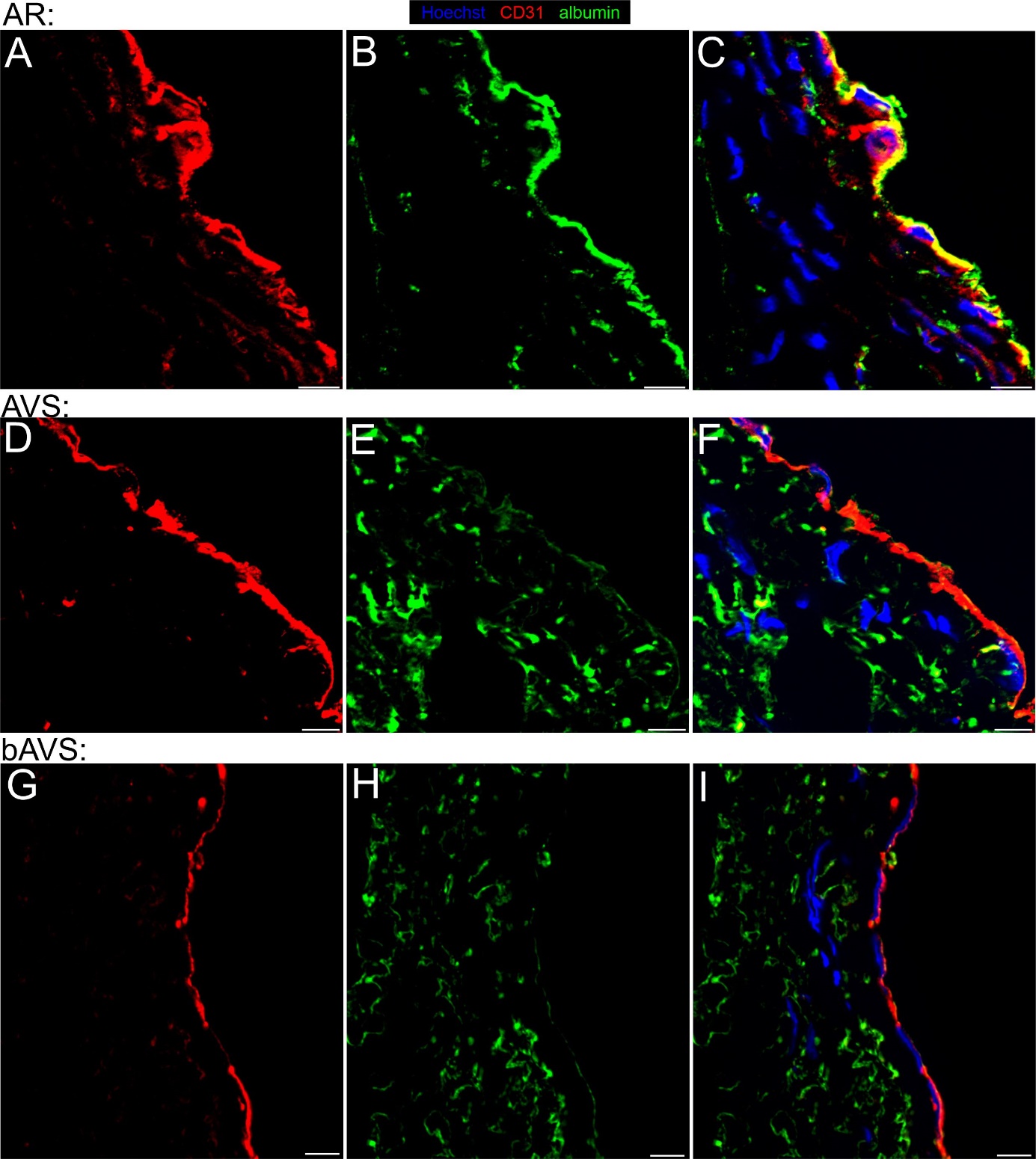


***Supplemental Figure 3: Endothelial barrier function in calcified and non-calcified aortic valves:*** *Stainings of aortic valves for CD31 (red) and albumin (green) in non-calcified ARs (****A-C****), AVS (****D-F****), and bAVS* ***(G-I)****; nuclei were stained with Hoechst. Scale bar = 10 µm.*


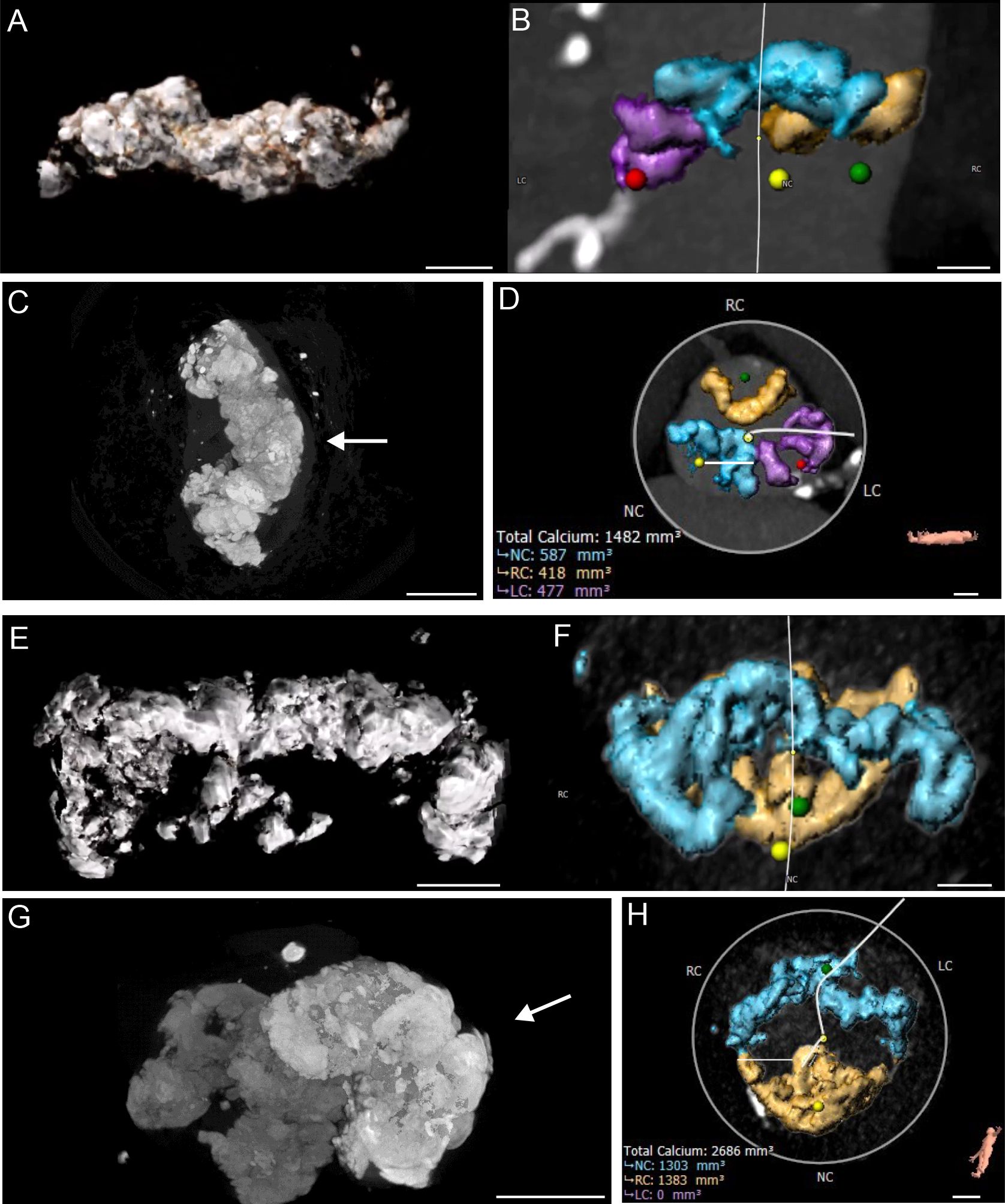


***Supplemental Figure 4: Clinical CT and Micro-CT scans of a calcified AVS and a calcified bAVS obtained before and after surgical valve removal****.* ***A,B****: Micro-CT (****A****) and CT (****B****) scans of a calcified AVS; the valve leaflet imaged in the micro-CT is labeled blue in the CT-scan.* ***C,D****: Micro-CT 3D reconstruction of the leaflet shown in* ***A*** *(arrow marks the aortic side) (****C****), CT image of all 3 leaflets of the AVS from the top view (****D****).* ***E,F****: Micro-CT (****E****) and CT (****F****) scans of a calcified bAVS; the valve leaflet imaged in the Micro-CT is labeled blue in the CT-scan.* ***G,H****: Micro-CT 3D reconstruction of the leaflet shown in* ***E*** *(arrow marks the aortic side) (****G****), CT image of all 2 leaflets of the bAVS from the top view (****H****). Scale bars= 5 mm.*

**Legends for supplemental videos**:

Video 1 (Supplemental Figure 4 A,B): Micro-CT and CT scans of a calcified AVS; the Micro-CT leaflet shown is labeled blue in the CT-scan;

Video 2 (Supplemental Figure 4 C): 3D reconstruction of the calcified AVS leaflet from Micro-CT;

Video 3 (Supplemental Figure 4 E,F): Micro-CT and CT scans of a calcified bAVS; the Micro-CT leaflet shown is labeled blue in the CT-scan;

Video 4 (Supplemental Figure 4 G): 3D reconstruction of the calcified AVS leaflet from Micro-CT;
